# DeepSom: a CNN-based approach to somatic variant calling in WGS samples without a matched normal

**DOI:** 10.1093/bioinformatics/btac828

**Published:** 2023-01-13

**Authors:** Sergey Vilov, Matthias Heinig

**Affiliations:** Institute of Computational Biology, Computational Health Center, Helmholtz Zentrum München Deutsches Forschungszentrum für Gesundheit und Umwelt (GmbH), 85764 Neuherberg, Germany; Institute of Computational Biology, Computational Health Center, Helmholtz Zentrum München Deutsches Forschungszentrum für Gesundheit und Umwelt (GmbH), 85764 Neuherberg, Germany; Department of Computer Science, TUM School of Computation, Information and Technology, Technical University Munich, 85748 Garching, Germany; DZHK (German Centre for Cardiovascular Research), Munich Heart Association, Partner Site Munich, 10785 Berlin, Germany

## Abstract

**Motivation:**

Somatic mutations are usually called by analyzing the DNA sequence of a tumor sample in conjunction with a matched normal. However, a matched normal is not always available, for instance, in retrospective analysis or diagnostic settings. For such cases, tumor-only somatic variant calling tools need to be designed. Previously proposed approaches demonstrate inferior performance on whole-genome sequencing (WGS) samples.

**Results:**

We present the convolutional neural network-based approach called DeepSom for detecting somatic single nucleotide polymorphism and short insertion and deletion variants in tumor WGS samples without a matched normal. We validate DeepSom by reporting its performance on five different cancer datasets. We also demonstrate that on WGS samples DeepSom outperforms previously proposed methods for tumor-only somatic variant calling.

**Availability and implementation:**

DeepSom is available as a GitHub repository at https://github.com/heiniglab/DeepSom.

**Supplementary information:**

Supplementary data are available at *Bioinformatics* online.

## 1 Introduction

Somatic variants (synonymously called here *mutations*) are genetic alterations accumulating in non-germline cells during the lifetime of the organism. Somatic mutations that promote cancer development by providing a selective growth advantage to the tumor are often referred to as driver mutations (Pon and Marra, 2015). All the other somatic mutations are termed passenger mutations. Driver mutations can corrupt genes regulating cell growth, programmed cell death, DNA repair pathway or neovascularization (Gao *et al.*, 2014; Hanahan and Weinberg, 2000; Li *et al.*, 1992; Reilly *et al.*, 2019; Young *et al.*, 2013). On the other hand, the accumulation of passenger mutations can slow down tumor growth and reduce metastatic progression (McFarland *et al.*, 2013, 2014). Therefore, identifying driver as well as passenger mutations is important to understand cancer genesis, choose treatment strategies and make prognosis.

Driver and passenger mutations can be represented by single nucleotide polymorphism (SNP) variants, short insertion and deletion (INDEL) variants, large copy number alterations and structural rearrangements (Ciriello *et al.*, 2013). In this article, we consider calling somatic SNP and short INDEL variants from short-read whole-genome sequencing (WGS) data, without distinguishing between drivers and passengers.

Technically, somatic variant calling should accomplish two tasks. First, true variants should be separated from sequencing and alignment artifacts. Second, somatic variants should be distinguished from germline mutations.

In popular somatic variant calling pipelines, such as GATK (McKenna *et al.*, 2010) and Strelka (Saunders *et al.*, 2012), these tasks are accomplished by constructing a joint alignment of reads from the tumor sample and its matched normal and then removing non-somatic variants using statistical models and specific filtering rules. In recent years, it has been shown that better results can be achieved using machine learning techniques.

For example, the method called Cerebro (Wood *et al.*, 2018) is based on extremely randomized trees that are trained on two sets of features derived from two tumor-normal alignments with two different alignment programs. Cerebro has been reported to detect somatic variants with a better accuracy compared to conventional callers. Deep learning-based methods, such as NeuSomatic (Sahraeian *et al.*, 2019) and DeepSSV (Meng *et al.*, 2021), have also demonstrated encouraging results.

However, a matched normal is not always available. This is a common scenario in a retrospective analysis of samples from clinical trials, pathology archives and legacy biobanks. Absence of a patient’s consent or financial restrictions can also hinder collection of a normal sample.

Removing artifacts in somatic variant calling without a matched normal is similar to removing artifacts when calling germline variants. Such filtering can be done based on various features. For example, some variants can first be removed based on their quality scores, then the remaining variants can be classified based on their variant allele fraction (VAF): germline variants are expected to have VAF around 50% (heterozygous) or 100% (homozygous), whereas artifacts usually have lower VAF. Although statistical models based on variant annotations have long been used for artifact filtering in germline variant calling (McKenna *et al.*, 2010; Saunders *et al.*, 2012), some recent studies have argued that machine learning techniques can provide a superior filtering quality.

For example, in the pioneering Deep Variant approach (Poplin *et al.*, 2018), candidate variants in the form of piled-up read images were presented to a convolutional neural network (CNN). The CNN output was then used to judge whether the input alteration was a true germline variant or an artifact. Deep Variant was reported to outperform filtering tools proposed in conventional germline variant calling pipelines GATK (McKenna *et al.*, 2010) and Strelka (Saunders *et al.*, 2012). Later on, an even better performance was achieved for an alternative CNN architecture and a slightly different variant encoding scheme (Friedman *et al.*, 2020).

In contrast to germline mutations, somatic variants often have a lower VAF due to tumor-normal contamination or tumor heterogeneity (Xu, 2018). This makes it more difficult to separate somatic mutations from artifacts. In this regard, highly accurate statistical modeling and advanced error correction techniques are of great importance.

Separating somatic and germline variants when a matched normal is not available is also challenging. Since candidate variants cannot be tested against a control sample, one can judge about a given variant only by comparing its characteristics with a priori information about somatic and germline mutations.

Current approaches to somatic variant calling without a matched control are represented by unsupervised and supervised methods (Supplementary Table S1).

Unsupervised methods usually classify mutations through modeling the germline VAF distribution for a given coverage in a given copy number segment. Such methods do not require a training set of labeled variants, use predefined filtering thresholds and do not consider disease-specific information.

SomVarIUS (Smith *et al.*, 2016) is one of the first unsupervised methods for tumor-only somatic variant calling. To separate somatic and germline variants, it builds for each candidate variant a beta-binomial model of the germline VAF distribution in the neighborhood. The model is fitted on variants that also appear in the dbSNP database and, therefore, likely to be germline. Artifacts are filtered out using a second statistical model which relies on base quality scores. The minimal recommended coverage for SomVarIUS is around 100×, which is above typical values used in routine WGS experiments (Björn *et al.*, 2018). Another unsupervised approach, named SGZ (Sun *et al.*, 2018), also estimates the germline VAF profile for each copy number segment, but does not use public variant databases to identify likely germline variants. LumosVar (Halperin *et al.*, 2017) additionally uses unmatched normal samples to label positions where the germline VAF distribution does not appear diploid in order to filter out potential mapping artifacts. The UNMASC pipeline (Little *et al.*, 2021) applies mixture models to identify hard-to-map (H2M) regions, filter out strand bias and sample preparation artifacts and eventually single out somatic mutations. VAF-based detection of copy number segments and classification methods built around precise VAF modeling require a very high read depth (Halperin *et al.*, 2017), so the effectiveness of SGZ, LumosVar and UNMASC was demonstrated on samples with average coverage of around 400×–800×, which is far above typical WGS values of around 30× (Björn *et al.*, 2018).

Compared to unsupervised techniques, supervised methods rely on specific features of a given tumor type, such as the characteristic somatic VAF and the somatic mutational signature (Alexandrov *et al.*, 2013). They may extensively use annotations derived from variant databases, such as COSMIC (Tate *et al.*, 2019), and variant effect prediction tools, such as snpEff (Cingolani *et al.*, 2012). The relevant disease-related statistics and specific variant filtering parameters are learnt in the training phase, which requires a set of labeled data (actual variant calls and/or alignment files) corresponding to a given tumor type.

For example, the supervised method called TOBI (Madubata *et al.*, 2017) uses gradient boosting to classify exonic somatic and germline variants based on VAF, variant annotation from COSMIC, variant effect prediction from snpEff, etc. Another supervised method, called ISOWN (Kalatskaya *et al.*, 2017), additionally uses somatic mutational signatures and information about flanking germline variants. Mutational signatures are imprints of particular DNA damage and repair mechanisms, specific for a given type of cancer and different from the spectrum of germline mutations (Alexandrov *et al.*, 2013). Flanking variants help to correct for shifts in VAF distributions due to local copy number variations. Similarly to TOBI, ISOWN can only classify somatic and germline SNP variants and does not propose any concrete strategy to filter out artifacts. In addition, none of these two methods was validated on WGS variants that are poorly represented in public cancer variant databases and whose effects are harder to predict.

In this study, we present DeepSom—a new pipeline for identifying somatic SNP and short INDEL variants in tumor WGS samples without a matched normal. DeepSom can effectively filter out both artifacts and germline variants under conditions of a typical WGS experiment. In the core of the method is a CNN trained on 3D tensors of piled-up variant reads. Using five different cancers datasets, we demonstrate that DeepSom can effectively filter out germline variants as well as artifacts. We show that such features as mutational signatures and flanking variants are important for correct variant classification. In addition, we find that DeepSom is robust with respect to potential mapping artifacts. Finally, we show that DeepSom outperforms two previously proposed methods for tumor-only somatic variant calling, namely SomVarIUS (Smith *et al.*, 2016) and ISOWN (Kalatskaya *et al.*, 2017).

## 2 Materials and methods

### 2.1 Input data

DeepSom has been trained and evaluated separately on each of five datasets representing five different cancers: TCGA-LAML (acute myeloid leukemia), BLCA-US (bladder urothelial cancer), ESAD-UK (esophageal adenocarcinoma), LINC-JP (liver cancer) and GACA-CN (gastric cancer). These datasets were downloaded from the ICGC portal (Zhang et al., 2019; ICGC, 2010) (https://dcc.icgc.org) and the TCGA portal (https://portal.gdc.cancer.gov).

Train/evaluation data for each dataset was generated based on tumor WGS samples (one BAM file per patient). Out of 43 WGS samples available for TCGA-LAML, one was excluded since its coverage was about 300×, far beyond typical read depth in WGS experiments. The total number of tumor WGS samples used, the number of somatic SNP and INDEL variants per sample and the median tumor coverage are shown in Table 1 for each dataset. VAF distributions are demonstrated in Supplementary Figure S1. Mutational signatures of somatic SNPs are shown in Supplementary Figure S2. Supplementary Figure S3 illustrates mutational signatures of artifacts and germline SNPs.

**Table 1.** Datasets considered in this study: total number of tumor WGS samples considered, average number of somatic SNPs per sample, average number of somatic INDELs per sample, and median coverage

Dataset	Tumor WGS samples	Somatic SNPs per sample	Somatic INDELs per sample	Median coverage
GACA-CN	25	11 451	505	33×
BLCA-US	23	22 055	705	34×
ESAD-UK	41	28 500	1739	59×
LINC-JP	28	9875	863	50×
TCGA-LAML	42	397	21	28×

To obtain training and evaluation data for each dataset, we first ran the Mutect2 caller (Benjamin *et al.*, 2019) (bundled with GATK v4) for each WGS tumor sample without a matched normal using default settings. In this regime, Mutect2 outputs all candidate variants, including true somatic and germline variants as well as sequencing and mapping artifacts.

To identify somatic mutations in the Mutect2 output, we downloaded lists of pre-called somatic variants from the corresponding resource. For ICGC samples, we considered consensus VCF files from the ICGC portal. From each consensus VCF file, we selected only variants on which all callers agreed. For the TCGA-LAML dataset, we considered somatic variants reported in Cancer Genome Atlas Research Network (2013). Although identifying germline variants in the Mutect2 output was not necessary to train and evaluate DeepSom, we marked them for further interpretation of CNN performance. To mark germline variants in ICGC samples, we downloaded the corresponding germline VCF files from the ICGC portal. Since germline variants for TCGA-LAML samples were not published, we called them ourselves with the GATK v.4 pipeline for germline short variant discovery (McKenna *et al.*, 2010) using variant quality score recalibration according to GATK Best Practices recommendations (DePristo *et al.*, 2011; Van der Auwera *et al.*, 2013).

### 2.2 gnomAD filtering

A single Mutect2 run on a WGS sample generates about 3.9–7.1 million candidate mutations, including about 3.0–5.4 million germline variants. The number of somatic variants is usually far lower (Table 1). While read qualities and read flags can be employed to filter out artifacts, they can hardly help to distinguish somatic and germline variants. Instead, VAF profiles (Supplementary Fig. S1) and mutational signatures (Supplementary Figs S2 and S3) can be used. However, since mutational signatures and VAF distributions for somatic and germline variants overlap, these features cannot ensure a perfect class separation. Hence, classification might be improved by supplying prior information. For example, it has been proposed (Kalatskaya *et al.*, 2017) to label candidate variants that are also encountered in public databases before running a machine-learning classifier.

**Fig. 1.**
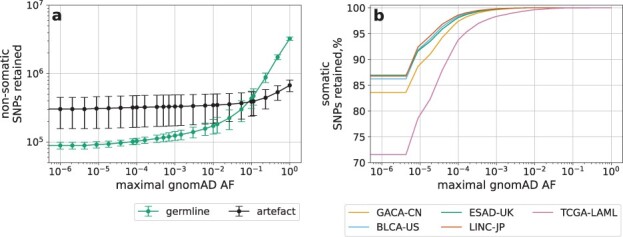
(**a**) The number of germline and artifact SNPs retained at different gnomAD allele frequency (AF) cutoffs. The errorbars show the standard deviation over all samples in all datasets. (**b**) The fraction of somatic SNPs retained at different gnomAD AF cutoffs in each dataset

In the present study, we consider the pre-filtering of candidate variants using the gnomAD v.3.1.2 database (Karczewski *et al.*, 2020), a collection of more than 500 million germline variants. Choosing the maximal gnomAD population allele frequency (AF) permits tuning the number of variants to exclude. Figure 1 shows the number of germline and artifact SNPs and the fraction of somatic SNPs retained at different gnomAD AF cutoffs. The corresponding plot for INDELs is demonstrated in Supplementary Figure S4.

To achieve the greatest reduction in the germline-to-somatic counts ratio, DeepSom excludes by default all gnomAD variants.

### 2.3 Tensor encoding

Before presented to the CNN, candidate variants were encoded following the approach in (Friedman *et al.*, 2020).

First, all reads covering the variant site were selected from the BAM file. Unmapped reads, reads that fail platform/vendor quality checks, PCR or optical duplicates were removed. Each read was then converted to a matrix of size *C *×* L*, where *C *=* *14 is the number of channels encoding the reference sequence and read features, *L* is the read length. The *C *=* *14 channels were composed of: 4 channels for one-hot encoding of the reference sequence, 4 channels for p-hot encoding of read base qualities (Friedman *et al.*, 2020), 6 channels for binary encoding of read flags (same for all positions in a given read). The following read flags were considered: read mapped in proper pair (0 × 2), mate unmapped (0 × 8), read reverse strand (0 × 10), mate reverse strand (0 × 20), not primary alignment (0 × 100) and supplementary alignment (0 × 800).

Afterwards, the reads were piled up around the variant site and stacked as a tensor with dimensions *C *×* W *×* *DP, where DP is the read depth, *W* is the length of the sequence region of interest (ROI) around the variant, the variant column being placed in the middle of ROI (for INDELs, the variant column corresponded to the position of the first inserted or deleted base). We chose *W *=* *150 as a further increase did not lead to any significant performance improvement (Supplementary Fig. S5). For each *k*bp-long insertion variant, we inserted a *k*-long sequence of deletions into the reference sequence (all zeros in one-hot encoding).

While the DP may not be the same for all variants, the CNN input should have predefined dimensions. So, we imposed the tensor height of *H *=* *70 (around the 80th percentile of read depth distribution for most datasets). Tensors with DP* *<* H* were padded to the full height *H* using extra reads with all channels filled with zeros. From tensors with DP* *>* H*, we removed DP–*H* excessive reads s.t. the resulting VAF was as close as possible to its initial value, providing the resolution of 1/*H*. The initial VAF and DP were saved for further use. Finally, all reads in the tensor were sorted by the base in the variant column (Friedman *et al.*, 2020).

### 2.4 Flanking regions

While the average VAF of germline heterozygous variants in the whole sample is around 50%, local deviations from this value may exist due to copy number alterations. This could lead to an inferior performance of a classifier which considers only the global difference between somatic and germline VAF distributions.

Local variations of germline VAF can be taken into account by using information about so-called flanking variants (Kalatskaya *et al.*, 2017). More precisely, for heterozygous variants, if the VAF of a germline variant to the left and the VAF of another germline variant to the right of the candidate variant is close to the VAF of the candidate variant, this candidate variant is more likely to be germline.

Similarly to ISOWN, we looked for flanking germline variants within a 4* *Mbp window around the candidate variant. At most two flanking variants on each side were considered. A given variant was considered germline if its gnomAD AF was above 10%.

Annotation for flanking variants was performed before gnomAD filtering.

### 2.5 CNN model

**Fig. 2.**
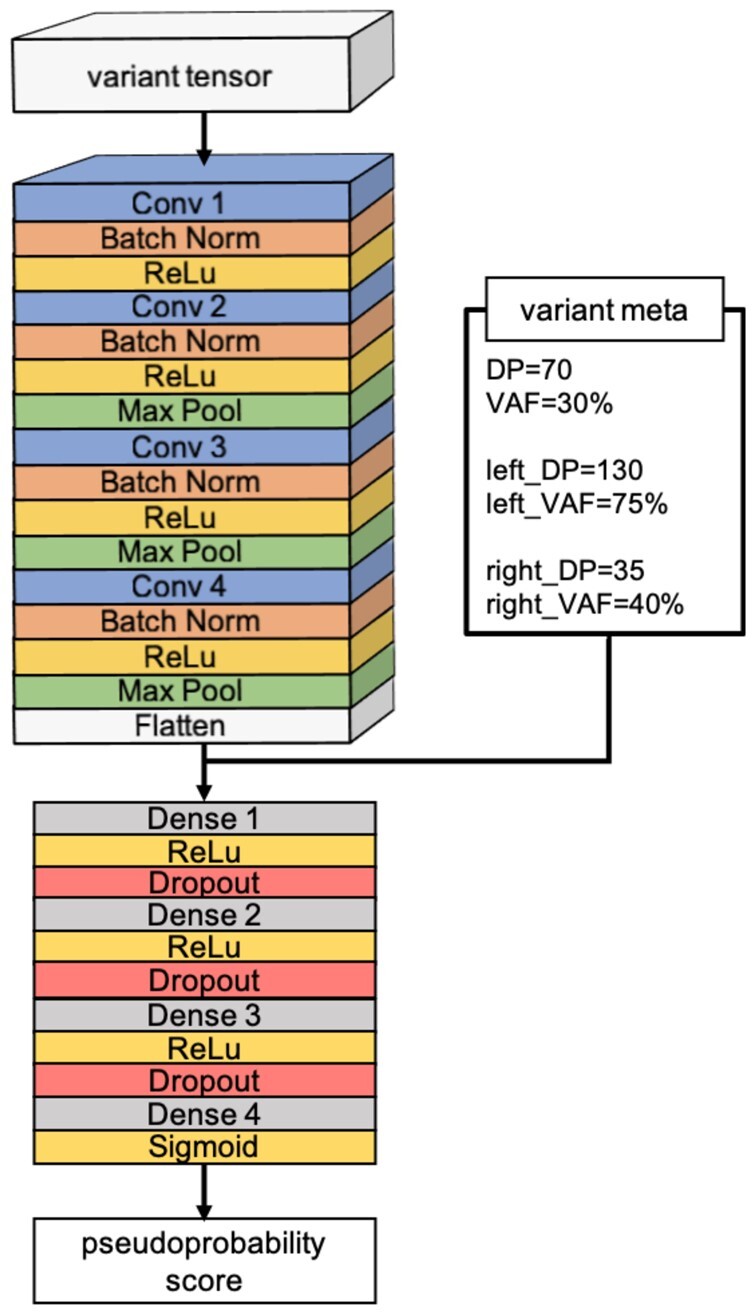
CNN architecture. Each variant is represented by a variant tensor and variant meta-information, the latter being added explicitly to the output of the convolutional block. The output of a sigmoid activation function in the final layer provides a pseudoprobability score that can be used to classify variants

The CNN model architecture is shown in Figure 2. The neural network prediction is based on the variant tensor and variant meta-information. The variant tensor is piped into a convolutional block which includes four convolutional layers. The output of the convolutional block is flattened out and concatenated with the meta-information. The concatenation is followed by four fully connected (dense) layers. The output of a sigmoid activation function after the final layer provides a pseudoprobability score that can be used to classify variants.

Variant meta-information includes 10 values: the VAF and DP for the candidate variant itself and for its two left and two right flanking variants. Whenever the variant tensor is cropped, we use VAF and DP computed before cropping.

The CNN model is implemented with the PyTorch framework (Paszke *et al.*, 2019). Optimization of model parameters in training is assured by the AdamW algorithm (Loshchilov and Hutter, 2018). At each training iteration, mini-batches of 32 variant tensors are presented to the CNN and the gradient of the binary cross-entropy loss function with respect to the model parameters is computed. The model parameters are then updated based on the computed gradient, according to the AdamW rules. After the first 15 epochs, the learning rate is reduced by a factor of 10 and the learning continues for 5 more epochs.

### 2.6 Performance metric

To evaluate the CNN performance, we compute the area under receiver operating characteristic curve (ROC AUC). The ROC AUC score does not depend on the ground truth class ratio in the dataset (Fawcett, 2006) and corresponds to the probability that a randomly chosen positive sample is ranked higher than a randomly chosen negative sample. Based on ROC AUC, model performance on datasets with different class ratios or on datasets of different variant types (e.g. SNPs and INDELs) can be easily compared.

The ROC curve plots the true positive rate (TPR), or recall, as a function of the false positive rate (FPR) for a series of CNN output thresholds (cutoffs), s.t. all variants with a CNN score above a given cutoff are classified as somatic. The TPR is defined as the percentage of total ground truth somatic variants *after* gnomAD filtering classified by the CNN as somatic, the FPR being the percentage of total ground truth non-somatic variants *after* gnomAD filtering classified as somatic.

While useful for CNN model selection and performance analysis, ROC AUC is not always the most appropriate metric for application purposes. To evaluate the performance of the entire DeepSom pipeline (gnomAD filtering+CNN), we compute the f1-score. By maximizing the f1-score, one tries to detect as many somatic variants as possible while keeping the number of false positives low. Calculating the f1-score involves choosing a particular CNN output cutoff. We choose the cutoff providing the highest f1-score. In particular, within each dataset we first take the median over the optimal thresholds determined for each sample individually, then we recompute each sample’s f1-score using this median as a new threshold value.

The f1-score is defined as the harmonic mean of precision and recall at a given CNN output cutoff: f1* *=* *2* *×* *precision* *×* *recall/(precision* *+* *recall), where the recall is the percentage of total ground truth somatic variants *before* gnomAD filtering classified by the CNN as somatic, while the precision is the fraction of true somatic variants among those classified as somatic. Separate cutoffs are used when DeepSom performance on somatic versus non-somatic, somatic versus germline and somatic versus artifact classification is reported.

For both ROC AUC and f1-score, we provide the average per-patient value, the reported errors show the standard deviation over different WGS samples (patients). Samples with less than 10 positive or less than 10 negative labels are excluded from performance assessment.

### 2.7 Training and evaluation

We evaluated DeepSom performance via 5-fold cross-validation (CV). The split was performed on the WGS sample (patient) level. In each CV round, each individual patient from the test fold was treated as an independent test dataset with positive (somatic variants) and negative (non-somatic variants) instances. The average and the standard deviation of the performance metric over all patients in all test folds were then reported.

In each CV round, the CNN was trained on tensors generated from 120k SNP (60k somatic and 60k non-somatic) and 20k INDEL (10k somatic and 10k non-somatic) variants randomly selected after gnomAD filtering from WGS samples chosen for training. Whenever the actual count of somatic variants in the training set was below the required number, upsampling was performed. In machine learning, using a balanced training set is a common way to avoid the model ignoring the minority class in the case of a substantial class imbalance. In particular, we observed that when trained without upsampling, the model would predict all instances as non-somatic in TCGA-LAML, where the number of ground truth somatic variants is the lowest (Table 1). Note that original unbalanced data were used as the test set on which performance is reported in order to get an objective and realistic performance assessment.

Further increase in the number of training variants did not lead to any significant performance improvement (Supplementary Fig. S6).

The average CNN training time was about 2.5 h on NVIDIA Tesla V100S, with the peak RAM allocation of around 1.5 Gb. The average inference time was around 30 min per WGS sample.

CNN hyper-parameter tuning was performed by maximizing ROC AUC via a random search (Bergstra and Bengio, 2012) within the first CV fold. The search range of hyper-parameters and their final values are summarized in Supplementary Table S2.

### 2.8 Running third-party variant calling tools

For DeepSom benchmarking, we chose SomVarIUS (Smith *et al.*, 2016; https://github.com/kylessmith/SomVarIUS) and ISOWN (Kalatskaya *et al.*, 2017; https://github.com/ikalatskaya/ISOWN), representing unsupervised and supervised approaches to tumor-only somatic variant calling correspondingly. SomVarIUS and ISOWN were run according to the guidelines in their official GitHub repositories.

SomVarIUS was run directly on each tumor BAM file for each dataset. All variants with SomVarIUS output *P*-value below the threshold were labeled somatic. This threshold was optimized with respect to the f1-score, in agreement with Section 2.6.

ISOWN performance on each dataset was evaluated via 5-fold CV using the same train/test splits as for DeepSom. In each CV fold, all variants labeled as artifacts (Section 2.1) were removed from the Mutect2 output and the remaining germline and somatic SNPs were presented for training and evaluation. ISOWN uses a Naive Bayes classifier and applies thresholding internally, s.t. it yields a list of putative somatic variants without reporting any probability-like score for each, which hinders optimization for a desired metric. Hence, we computed the f1-score directly on ISOWN output, as done in the original work (Kalatskaya *et al.*, 2017).

To be consistent with the published SomVarIUS and ISWON workflows, no gnomAD filtering was applied when running these two methods. Additionally, using gnomAD-filtered data would make it difficult for SomVarIUS and ISOWN to take into account local variations of the germline VAF distribution as potential flanking germline variants might be filtered out.

### 2.9 Annotation of H2M regions

It was previously reported (Halperin *et al.*, 2017; Little *et al.*, 2021) that variant calls might be unreliable in regions of high mapping uncertainty, so-called H2M regions.

We consider a variant being in a H2M region if it belongs to at least one of the four categories: (i) resides inside a repeat, (ii) resides in a highly polymorphic region represented by a genetic variant hotspot (Long and Xue, 2021; Supplementary Dataset S1), (iii) has a UMAP (Karimzadeh *et al.*, 2018) mappability score below 1.0 and (iv) resides inside a H2M gene (Little *et al.*, 2021). Repeats were annotated using the RepeatMasker UCSC track (https://genome.ucsc.edu/cgi-bin/hgTrackUi?g=rmsk). The variant was considered belonging to a repeat if it was placed inside the repeat more than the read length apart (75 bp for TCGA-LAML and 100 bp for the other datasets) from the repeat edge. The UMAP hg19 multi-read mappability track was derived from the official project page (https://bismap.hoffmanlab.org/). As H2M genes, we considered all genes that were fully or partially detected as H2M by UNMASC (Little *et al.*, 2021). To each variant, the corresponding gene label was assigned using the snpEff variant annotation toolbox v.5.0e (Cingolani *et al.*, 2012).

The percentage of somatic and non-somatic variants in each H2M region is shown in Supplementary Table S3.

### 2.10 Mutational signatures

Mutational signatures of SNP variants were constructed by computing the probability of each possible mutation, the mutation being defined as the 3 bp-window of the reference sequence around the variant site plus the alternative allele (Alexandrov *et al.*, 2013). For each dataset, this probability was computed as the percentage of a given mutation in a pool of all mutations of the corresponding variant type (somatic, germline or artifact). Mutation signatures were further normalized using the re-occurrence of each mutation in the reference genome.

## 3 Results

**Fig. 3.**
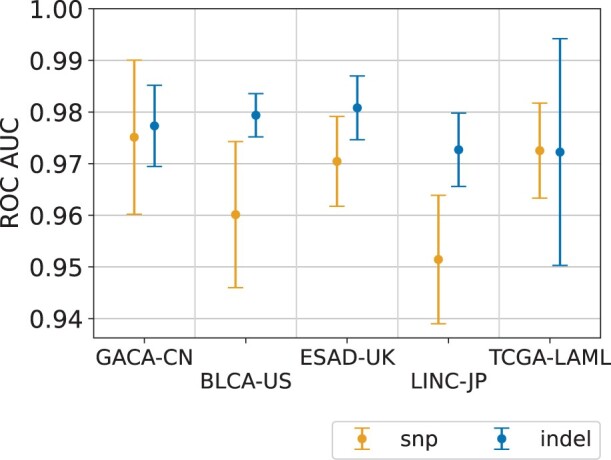
CNN performance on SNP and INDEL variants. The average ROC AUC score is 0.966 ± 0.009 and 0.976 ± 0.003 on SNP and INDEL classification correspondingly

CNN performance on SNP and INDEL variants is illustrated in Figure 3. The ROC AUC score remains high across all datasets, with the average values being 0.966 ± 0.009 and 0.976 ± 0.003 on SNP and INDEL classification correspondingly. The errorbars indicate that the model generalizes well across different samples of the same cancer type. The corresponding ROC curves for individual samples are shown in Supplementary Figure S7.

**Fig. 4.**
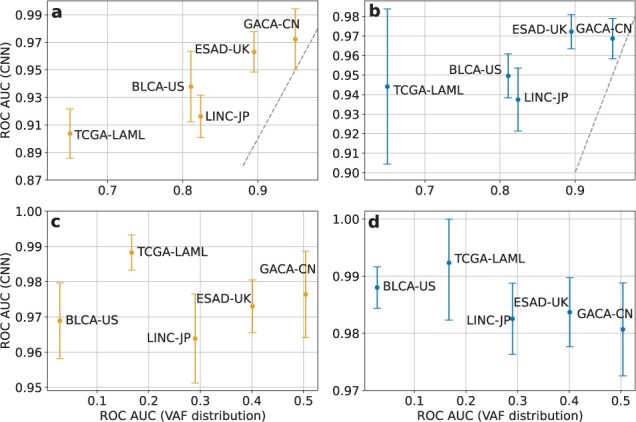
CNN ROC AUC score on somatic versus germline SNP (**a**) and INDEL (**b**) classification and on somatic versus artifact SNP (**c**) and INDEL (**d**) classification as a function of the ROC AUC score of a simple VAF-driven classifier. The dashed gray line in (a) and (b) corresponds to the equivalent performance of the CNN and the VAF-driven classifier. The CNN outperforms the VAF-driven classifier and scores better in somatic versus artifact classification than in somatic versus germline classification. In somatic versus germline classification, the CNN performance and the performance of the VAF-driven classifier are correlated

To assess how well the CNN removes artifacts and how well it filters out germline variants, we computed ROC AUC scores separately on somatic versus artifact classification and on somatic versus germline classification. These scores were then compared to the performance of a simple VAF-driven variant filtering method that rejects all variants whose VAF is above (below) a value chosen on VAF distributions (Supplementary Fig. S1). The results are shown in Figure 4.

As follows from Figure 4, the CNN greatly outperforms the simple VAF-based filtering method and effectively removes artifacts as well as germline variants, with a better performance in somatic versus artifact classification. The performance difference between somatic versus artifact and somatic versus germline classification is likely due to these two classifications relying on overlapping but different sets of features: read quality scores might play a greater role in artifact filtering whereas VAF might be more important when eliminating true germline variants. Note that the CNN score in Figure 4a and b is correlated with the performance of the simple VAF-driven filtering method, which is in turn determined by the difference between VAF distributions of somatic and germline variants. This indicates an important role of VAF in somatic versus germline classification. On the other hand, artifact filtering seems independent of VAF distributions (Fig. 4c and d).

To assess the effect of mutational signatures on classification, we trained the CNN while replacing mutational signatures of somatic SNP variants with randomly chosen mutational signatures of non-somatic SNP variants. The ROC AUC scores on SNP classification for models trained with and without signature replacement are compared in Supplementary Table S4.

Supplementary Table S4 demonstrates that the CNN performs better when mutational signatures of somatic and non-somatic variants differ. The largest ROC AUC increase is observed for BLCA-US, LINC-JP and ESAD-UK, followed by GACA-CN and TCGA-LAML. Note that the difference between somatic and germline mutational signatures is the highest for ESAD-UK and the lowest for TCGA-LAML (Supplementary Fig. S8). We also note that not only true variants but also artifacts have their own mutational signatures that were observed to be stable within the same sequencing protocol (Supplementary Fig. S3). Hence, using mutational signatures might improve not only somatic versus germline classification but also somatic versus artifact classification.

To study further the role of mutational signatures in classification, we computed CNN saliency maps (Simonyan *et al.*, 2013). An example saliency map for a SNP variant is shown in Supplementary Figure S9(a–c). It can be seen that apart from the variant site itself, 5–10 neighboring bases are also involved in classification. In case of an INDEL variant, the number of relevant bases is close to the INDEL size [Supplementary Fig. S9(d–f)]. The further performance increase with the ROI length (Supplementary Fig. S5) can be related to a better filtering of mapping artifacts and classification of rare larger INDEL variants.

To assess the effect of flanking variants on classification, we trained the CNN when replacing VAF and read depth of flanking variants with zeros. For all the datasets, the loss of information about flanking variants degraded the performance (Supplementary Table S5).

To assess the CNN generalization, we performed cross-cancer evaluation by applying the model trained on each single dataset to all the other datasets. The results are shown in Table 2.

**Table 2.** CNN ROC AUC score in cross-cancer evaluation

Train dataset	Test dataset									
	GACA-CN		BLCA-US		ESAD-UK		LINC-JP		TCGA-LAML	
	SNP	INDEL	SNP	INDEL	SNP	INDEL	SNP	INDEL	SNP	INDEL
GACA-CN	0.975	0.977	0.923	0.955	0.927	0.961	0.906	0.947	0.957	0.964
BLCA-US	0.937	0.966	0.960	0.979	0.934	0.966	0.929	0.964	0.966	0.976
ESAD-UK	0.933	0.958	0.938	0.965	0.970	0.981	0.934	0.962	0.953	0.966
LINC-JP	0.930	0.958	0.938	0.973	0.947	0.972	0.951	0.973	0.970	0.976
TCGA-LAML	0.873	0.899	0.868	0.898	0.817	0.874	0.840	0.875	0.973	0.972

*Note*: When the train and test datasets match, 5-fold cross-validation is used, otherwise the model is trained on all WGS samples from the train dataset and tested on all WGS samples from the test dataset. All models demonstrate good generalization performance.

As can be seen from Table 2, the CNN mostly demonstrates good cross-cancer generalization despite differences between the VAF distributions (Supplementary Fig. S1) and the mutational signatures (Supplementary Fig. S2). One of the reasons is that read qualities are independent of the cancer type, so somatic versus artifact classification is partially cancer-independent. Additionally, the somatic VAF distribution is always shifted to the left with respect to the germline VAF distribution (Supplementary Fig. S1), so somatic versus germline classification is also partially generalizable. Somatic mutational signatures are, however, cancer-dependent, which degrades the generalization performance.

It is interesting to note that the model trained on TCGA-LAML shows the worst generalization performance whereas the models trained on the other datasets generalize on TCGA-LAML significantly better. A possible reason could be that TCGA-LAML somatic variants are of higher quality because they were validated in additional targeted sequencing experiments (Cancer Genome Atlas Research Network, 2013). Since high quality variants are easier to classify (Friedman *et al.*, 2020; Poplin *et al.*, 2018), models trained on other datasets perform remarkably well on TCGA-LAML. On the other hand, the TCGA-LAML model can distinguish only ‘easy’ variants, which explains its worse generalization performance.

To investigate how well DeepSom handles variants from H2M and non-H2M regions, we first tested the trained CNN models on regions of different mappability (Section 2.9). The ROC AUC score on the corresponding subgroups of test variants is shown in Supplementary Table S6. The score slightly improves on variants from non-H2M regions (+0.003 on SNPs and +0.004 on INDELs on average), while it slightly decreases on H2M regions (−0.001 on SNPs and −0.007 on INDELs).

To study how the presence of variants from H2M regions in the training set influences the CNN performance, we retrained the classifier excluding all variants belonging to at least one H2M region. As follows from Supplementary Table S7, training without mutations from H2M regions moderately improves the score on variants from non-H2M regions (+0.001 on SNPs and +0.003 on INDELs) while further decreasing the score on variants from H2M regions (−0.008 on SNPs and −0.004 on INDELs). This behavior is consistent with the concept of a machine learning classifier that should perform better on data statistically similar to the training set. It is worth mentioning that among different H2M categories, the worst performance is observed on variants with low UMAP scores (−0.038 on SNPs and −0.029 on INDELs compared to non-H2M), whereas variants in H2M genes were classified nearly as well as variants from non-H2M regions. The latter could be due to the chosen variants being outside H2M loci of the selected genes. The small magnitude of the improvement on variants from non-H2M regions indicates that DeepSom training is robust with respect to mapping artifacts.

To assess the biological significance of somatic variant calls provided by DeepSom, we computed the fraction of somatic and non-somatic variants in cancer gene census (CGC) genes (Sondka *et al.*, 2018) before and after classification. For this analysis, we chose only variants labeled as high-impact mutations by the snpEff toolbox v.5.0e (Cingolani *et al.*, 2012). The results are shown in Supplementary Table S8. In agreement with the ground truth data, DeepSom classification leads to a higher proportion of somatic variants in CGC genes (7.8% for SNPs and 8.6% for INDELs) compared to that of non-somatic ones (5.2% for SNPs and 6.3% for INDELs). The difference between the predicted somatic and non-somatic proportions is, however, somewhat smoothed out compared to the ground truth due to gnomAD filtering and imperfect class separation.

We also assessed DeepSom performance at different gnomAD AF filtering thresholds. Both the CNN ROC AUC score and DeepSom f1-score diminish at high gnomAD AF thresholds (Supplementary Fig. S10). The CNN performance drops due to the reduced difference between germline and somatic mutational signatures as measured by the Jensen-Shannon divergence (Supplementary Fig. S8), accompanied by the increased ratio between germline variants and artifacts (Fig. 1a and Supplementary Fig. S4a). The f1-score also decreases because the recall improvement due to less restrictive gnomAD filtering (Fig. 1b and Supplementary Fig. S4b) is not sufficient to compensate for the degraded CNN performance.

Finally, we also applied two methods previously proposed for somatic variant calling without a matched normal, specifically SomVarIUS and ISOWN, to all the datasets. Table 3 compares f1-score for DeepSom, SomVarIUS and ISOWN. The respecting DeepSom CNN cutoffs, precision and recall are shown in Supplementary Tables S9–S11.

**Table 3.** F1-score for DeepSom, SomVarIUS (Smith *et al.*, 2016), and ISOWN (Kalatskaya *et al.*, 2017) on somatic versus germline&artifact, somatic versus germline and somatic versus artifact classification

Dataset	DeepSom						SomVarIUS			ISOWN
	SNP			INDEL			SNP			SNP
	som versus germ&art	som versus germ	som versus art	som versus germ&art	som versus germ	som versus art	som versus germ&art	som versus germ	som versus art	som versus germ
GACA-CN	0.41	0.59	0.44	0.18	0.34	0.21	0.10	0.22	0.13	2.4e−3
BLCA-US	0.46	0.57	0.54	0.20	0.30	0.29	0.032	0.042	0.042	2.9e−3
ESAD-UK	0.56	0.68	0.60	0.28	0.45	0.33	0.14	0.24	0.16	1.8e−3
LINC-JP	0.32	0.42	0.42	0.19	0.30	0.25	0.025	0.049	0.029	2.5e−3
TCGA-LAML	0.032	0.048	0.089	0.038	0.044	0.39	1.0e−3	4.0e−3	1.7e−3	7.8e−3

*Note*: DeepSom generally outperforms SomVarIUS by an order of magnitude and ISOWN by two orders of magnitude.

As can be seen from Table 3, DeepSom usually outperforms SomVarIUS by an order of magnitude and ISOWN by two orders of magnitude. It is also worth mentioning that DeepSom precision and recall are generally of the same order of magnitude, SomVarIUS recall exceeds its precision, while ISOWN recall is far below its precision. The inferior performance of SomVarIUS can be due to using hard classification thresholds and its limited ability to learn directly from data. In addition, SomVarIUS completely ignores mutational signatures. On the other hand, ISOWN demonstrates an inferior performance presumably because it relies heavily on various cancer databases in which many WGS somatic variants are not represented: 97% of variants classified by ISOWN as somatic were found in exonic regions, about 50% of them also being in COSMIC. Due to its strong dependence on cancer variant databases, ISOWN demonstrates a relatively high precision (about 0.4 on average, Supplementary Table S10) accompanied by a very low recall (about 1.3e−3 on average, Supplementary Table S11).

We also note that DeepSom f1-scores exhibit higher sample-to-sample fluctuations (Supplementary Fig. S11) compared to ROC AUC (Fig. 3). These fluctuations stem from the highly variable number of ground truth somatic variants in tumor samples which leads to large variations in precision at a fixed CNN output threshold. High sample-to-sample oscillations of precision and f1-score were also reported previously for LumosVar (Halperin *et al.*, 2017) and UNMASC (Little *et al.*, 2021).

## 4 Discussion

DeepSom is a novel pipeline for somatic variant calling without a matched normal. DeepSom demonstrated a competitive performance on five different cancer datasets. These datasets have diverse VAF distributions (Supplementary Fig. S1) and mutational signatures (Supplementary Fig. S2 and S3) and thus represent different levels of ’difficulty’ for the caller. Note also that DeepSom performed well despite trained on the small number of WGS samples available for each dataset in the repository.

DeepSom uses WGS BAM files and the germline variant database gnomAD as the input. The DeepSom pipeline consists of three major steps: calling candidate variants, gnomAD pre-filtering and CNN-based classification.

Firstly, all possible variants (somatic, germline and artifacts) are called. The variant caller used at this step should be able to provide candidate variants without using a matched normal. Similarly to ISOWN, DeepSom makes use of the Mutect2 caller (Benjamin *et al.*, 2019). All the variants which are not identified at this step (e.g. due to internal Mutect2 filtering), will be permanently lost. If high sensitivity is required, Mutect2 can be bypassed by calling all positions with at least one read with an alternative allele.

Afterwards, the VCF files are annotated for flanking variants using the gnomAD database. It was previously reported (Little *et al.*, 2021) that germline variant databases might contain artifacts, which could potentially hinder the proper identification of flanking variants. In this regard, we note that DeepSom extracts flanking information based on gnomAD variants with a minimum population AF of 10%, which should lower the chance of hitting a gnomAD artifact. Additionally, one may consider masking likely artifacts when looking for flanking variants without necessarily removing them from the DeepSom inference pool. In particular, strand bias and sample preparation artifacts, such as FFPE and oxoG artifacts, can be labeled using GATK FilterByOrientationBias and FischerStrand tools (McKenna *et al.*, 2010), SOBDetector (Diossy *et al.*, 2021) or via UNMASC filtering (Little *et al.*, 2021).

The purpose of the next step is to remove a significant part of germline variants based on their presence in gnomAD. The reduction in germline variant count is achieved at the expense of a certain fraction of somatic variants (Fig. 1 and Supplementary Fig. S4). However, somatic variants present in gnomAD could well be harmless passenger mutations and, therefore, might often be safely neglected.

Finally, the CNN-based classifier generates a pseudoprobability score based on the variant data. The variant classification is then performed by placing a threshold on this pseudoprobability score. This threshold should be selected depending on a particular application. For example, a threshold maximizing the f1-score can be used for mutational load estimation whereas a threshold that guarantees a fixed TPR could be more appropriate when looking for novel mutations. Note that the reported f1-score thresholds (Supplementary Table S9) should be carefully considered in view of large sample-to-sample f1-score fluctuations (Supplementary Fig. S11).

In line with previous research (Little *et al.*, 2021), we demonstrated that disregarding H2M regions improves the classifier performance on variants from non-H2M regions. In view of the moderate magnitude of this performance gain and the more significant performance drop on variants from H2M regions, we do not recommend excluding H2M regions from the training set. Should H2M filtering be applied at the inference stage, it can be omitted for variants from predefined white lists, e.g. those from the COSMIC database (Little *et al.*, 2021). We admit, however, that our identification of H2M regions relies on genome-specific annotations and may ignore certain protocol-specific features. When a higher precision in H2M regions detection is required, VAF-based segmentation (Little *et al.*, 2021) can be used.

DeepSom surpasses previously proposed methods for tumor-only somatic variant calling, namely SomVarIUS and ISOWN, both qualitatively (Supplementary Table S1) and quantitatively. By using a more advanced CNN-based classifier and including read qualities and read flags information, DeepSom does not only outperform ISOWN in somatic versus germline classification but also provides a means for efficient artifact filtering. In contrast to both SomVarIUS and ISOWN, DeepSom can call not only SNP but also INDEL variants.

We believe that DeepSom might be particularly suitable for research purposes, e.g. discovering novel variants in retrospective studies using arrays of unmatched tumor samples. In clinical diagnostics, tumor-only sequencing is usually performed on a predefined gene panel with known recurrent mutations that are part of guidelines (Khoury *et al.*, 2022). In this case, DeepSom might be used as a complementary filtering tool to obtain higher quality calls with fewer artifacts. Alternatively, one may apply DeepSom as a part of a more elaborate variant calling pipeline, e.g. UNMASC (Little *et al.*, 2021).

We note that DeepSom performance is largely bound by the sequencing technology. To improve somatic versus artifact classification, a sequencer must produce more reliable base quality scores. On the other hand, using longer reads and more advanced aligners should reduce the number of mapping artifacts. To improve both somatic versus artifact and somatic versus germline classification, sequencing depth (coverage) should be increased, s.t. the error in VAF estimation is reduced. However, one should keep in mind that higher coverage comes at higher cost and may be associated with an increased number of artifacts (Chen *et al.*, 2020).

DeepSom could potentially be extended to a three-class problem, simultaneously classifying somatic versus germline versus artifact variants, by modifying the CNN architecture and changing the loss function accordingly. This extension would involve several additional challenges to overcome: one would need to provide reliable training/evaluation labels for all the three classes and to cope with the corresponding class imbalance. Note, however, that germline variants and artifacts filtered out by gnomAD do not take part in DeepSom inference. Therefore, this three-class model would remain more suitable for somatic variant detection. The current design of DeepSom already considers mutational context, VAF and read orientation-specific information encoded in the variant tensor, so DeepSom could potentially further classify detected artifacts into subclasses, including oxoG, FFPE or other strand bias artifacts provided that the corresponding ground truth labels are available at training.

## 5 Conclusion

In this work, we presented the CNN-based approach called DeepSom for identifying somatic variants in WGS samples without a matched normal. DeepSom largely outperforms the tested methods for tumor-only somatic variant calling and provides a way to call not only SNP but also INDEL variants. DeepSom does not require any manual tuning and can be applied under typical WGS experimental conditions. The proposed pipeline could potentially be used to discover novel somatic mutations in research setting, or in clinical practice when supported with other filtering tools.

DeepSom is freely available as a GitHub repository.

## Supplementary Material

btac828_Supplementary_Data

## Data Availability

All datasets except TCGA-LAML were derived from the ICGC portal: https://dcc.icgc.org. The TCGA-LAML dataset was derived from the TCGA portal: https://portal.gdc.cancer.gov. Somatic variants for the TCGA-LAML dataset were obtained from Supplementary Material to Cancer Genome Atlas Research Network (2013).
