# Supplementary material for: DeepSom: a CNN-based approach to somatic variant calling in WGS samples without a matched normal
Source: Bioinformatics. 2023 Jan 13;39(1):btac828. doi: 10.1093/bioinformatics/btac828 (PMC9843587; doi:10.1093/bioinformatics/btac828)
Supplement: btac828_Supplementary_Data [file btac828_supplementary_data.pdf]

Supplementary materials for

DeepSom: a CNN-based approach to somatic variant calling in WGS samples without a matched normal

Sergey Vilov[1] and Matthias Heinig[1,2]

[1]Institute of Computational Biology, Computational Health Center, Helmholtz Zentrum München Deutsches Forschungszentrum für Gesundheit und Umwelt (GmbH), 85764 Neuherberg, Germany

[2]Department of Informatics, Technical University Munich, Garching, 85748, Germany.

	Unsupervised			Supervised	
	SomVarIUS, SGZ	LumosVar	UNMASC	ISOWN TOBI	DeepSom
classifies SNPs	■	■	■	■	■
classifies INDELs	■	■	■		■
can remove sequencing artefacts	■	■	■		■
can remove strand bias artefacts			■		■
validated on WGS data					■

Table S1. Comparison of tumor-only somatic variant calling pipelines: SomVarIUS (Smith *et al.*, 2016), SGZ (Sun *et al.*, 2018), LumosVar (Halperin *et al.*, 2017), UNMASC (Little *et al.*, 2021), ISOWN (Kalatskaya *et al.*, 2017), TOBI (Madubata *et al.*, 2017), and DeepSom (this paper).

Hyper-parameter	Search space	Final value
Mini-batch size	[8, 16, 32, 64, 128, 256]	32
Learning rate	1e-8 … 1e-2	1e-3
Weight decay	1e-6 … 1e-1	1e-1
Dropout	0.1 … 0.7	0.5
Convolutional kernel size	[3, 5]	3
Num. of convolutional channels	[(16, 16, 16, 16), (16, 32, 16, 32), …]	(32, 32, 32, 32)
Num. of units in dense layers	[(256, 128, 64), (128, 64, 64), …]	(256, 256, 128)

Table S2. Search range and final values of CNN hyper-parameters.

variant class	dataset		H2M gene	low UMAP score	inside repeat	GV hotspot	any H2M region
somatic	GACA-CN	SNP	<1%	1%	19%	2%	21%
		INDEL	<1%	1%	25%	2%	27%
	BLCA-US	SNP	<1%	1%	21%	2%	23%
		INDEL	<1%	1%	23%	3%	25%
	ESAD-UK	SNP	<1%	1%	25%	3%	27%
		INDEL	<1%	1%	26%	3%	28%
	LINC-JP	SNP	<1%	1%	22%	2%	24%
		INDEL	<1%	1%	26%	3%	29%
	TCGA-LAML	SNP	<1%	1%	2%	3%	5%
		INDEL	<1%	2%	2%	3%	8%
	**average**	SNP	<1%	1%	18%	2%	20%
		INDEL	<1%	1%	20%	3%	23%
non-somatic	**average**	SNP	1%	27%	31%	7%	50%
		INDEL	1%	14%	19%	6%	33%

Table S3. Percentage of somatic and non-somatic variants in different H2M regions: *H2M gene* - only variants from H2M genes, *low UMAP score* - only variants with UMAP mappability score below 1, *inside repeat* - only variants in repeats, *GV hotspot* - only variants in genetic variant (GV) hotspots, *any H2M region* - variants belonging to at least one H2M region.

dataset	ROC AUC		paired t-test p-value
	original SNP signatures	replaced SNP signatures	
GACA-CN	0.975±0.015	0.970±0.018	<0.01
BLCA-US	0.960±0.014	0.941±0.013	<0.01
ESAD-UK	0.970±0.009	0.961±0.011	<0.01
LINC-JP	0.951±0.013	0.941±0.015	<0.01
TCGA-LAML	0.973±0.009	0.970±0.011	<0.01

Table S4. CNN performance on SNP variants when trained with original somatic SNP signatures and when trained on somatic SNP signatures replaced with non-somatic ones. The paired t-test p-values confirm that mutational signatures are relevant for classification.

dataset		ROC AUC		paired t-test p-value
		with flanking regions	without flanking regions	
GACA-CN	SNP	0.975±0.015	0.972±0.017	<0.01
	INDEL	0.977±0.008	0.975±0.009	<0.01
BLCA-US	SNP	0.960±0.014	0.952±0.018	<0.01
	INDEL	0.979±0.004	0.977±0.005	<0.01
ESAD-UK	SNP	0.970±0.009	0.959±0.012	<0.01
	INDEL	0.981±0.006	0.976±0.007	<0.01
LINC-JP	SNP	0.951±0.013	0.941±0.015	<0.01
	INDEL	0.973±0.007	0.967±0.008	<0.01
TCGA-LAML	SNP	0.973±0.009	0.969±0.012	<0.01
	INDEL	0.972±0.023	0.971±0.022	0.456

Table S5. CNN performance when trained with and without information about flanking variants. The paired t-test p-values confirm that flanking variants are relevant for classification.

dataset		all regions	without H2M	H2M gene	low UMAP score	inside repeat	GV hotspot	any H2M region
GACA-CN	SNP	0.975	0.976	0.987	0.948	0.975	0.977	0.976
	INDEL	0.977	0.979	-	-	0.970	0.967	0.973
BLCA-US	SNP	0.960	0.963	0.983	0.942	0.960	0.958	0.962
	INDEL	0.979	0.984	-	-	0.967	0.957	0.969
ESAD-UK	SNP	0.970	0.974	0.979	0.946	0.970	0.967	0.970
	INDEL	0.981	0.983	-	0.960	0.971	0.975	0.975
LINC-JP	SNP	0.951	0.955	0.971	0.924	0.953	0.951	0.950
	INDEL	0.973	0.979	-	-	0.959	0.968	0.964
TCGA-LAML	SNP	0.973	0.974	-	-	-	0.968	0.967
	INDEL	0.972	0.976	-	-	-	-	-

Table S6. CNN ROC AUC scores when trained on *all genomic regions* and tested on regions of different mappability: *all regions* - all variants (also Fig. 3, Table 1), *without H2M* - excluding variants belonging to at least one H2M region, *H2M gene* - only variants from H2M genes, *low UMAP score* - only variants with UMAP mappability score below 1, *inside repeat* - only variants in repeats, *GV hotspot* - only variants in genetic variant (GV) hotspots, *any H2M region* - variants belonging to at least one H2M region.

dataset		all regions	without H2M	H2M gene	low UMAP score	inside repeat	GV hotspot	any H2M region
GACA-CN	SNP	0.972	0.976	0.983	0.948	0.971	0.975	0.971
	INDEL	0.978	0.985	-	-	0.967	0.962	0.970
BLCA-US	SNP	0.957	0.965	0.977	0.934	0.954	0.955	0.954
	INDEL	0.978	0.985	-	-	0.964	0.956	0.965
ESAD-UK	SNP	0.964	0.975	0.968	0.931	0.961	0.961	0.960
	INDEL	0.980	0.986	-	0.957	0.966	0.974	0.970
LINC-JP	SNP	0.945	0.957	0.955	0.908	0.942	0.944	0.938
	INDEL	0.975	0.983	-	-	0.956	0.958	0.960
TCGA-LAML	SNP	0.972	0.975	-	-	-	0.961	0.960
	INDEL	0.972	0.977	-	-	-	-	-

Table S7. CNN ROC AUC scores when trained *only on non-H2M regions* and tested on regions of different mappability: *all regions* - all variants, *without H2M* - excluding variants belonging to at least one H2M region, *H2M gene* - only variants from H2M genes, *low UMAP score* - only variants with UMAP mappability score below 1, *inside repeat* - only variants in repeats, *GV hotspot* - only variants in genetic variant (GV) hotspots, *any H2M region* - variants belonging to at least one H2M region.

dataset		GROUND TRUTH					PREDICTED	
		non-somatic	non-somatic in CGC genes, %	somatic	somatic in CGC genes, %	Fisher's test p-value	non-somatic in CGC genes, %	somatic in CGC genes, %
GACA-CN	SNP	17324	2.4±0.2	93	15.1±7.4	<0.01	5.1	9.7
	INDEL	11444	3.8±0.4	23	21.7±17.8	<0.01	6.3	9.5
BLCA-US	SNP	16821	2.6±0.2	513	11.3±2.7	<0.01	6.2	8.4
	INDEL	12397	3.8±0.3	107	25.2±8.3	<0.01	6.7	14.0
ESAD-UK	SNP	29802	2.6±0.2	420	10.5±2.9	<0.01	5.1	4.8
	INDEL	20470	3.8±0.3	130	15.4±6.3	<0.01	6.4	5.9
LINC-JP	SNP	21833	2.7±0.2	178	7.9±4.0	<0.01	4.8	6.5
	INDEL	13792	3.8±0.3	115	6.1±4.4	0.21	6.1	0.0
TCGA-LAML	SNP	32078	3.3±0.2	32	46.9±18.0	<0.01	5.3	16.7
	INDEL	22651	4.5±0.3	23	56.5±21.4	<0.01	6.2	16.7
**total**	SNP	117858	2.8±0.1	1236	11.7±1.8	<0.01	5.2	7.8
	INDEL	80754	4.0±0.1	398	18.1±3.8	<0.01	6.3	8.6

Table S8. Proportions of somatic and non-somatic (germline+artefacts) variants in cancer gene census (CGC) genes after selecting only variants with a 'HIGH' impact (according to snpEff annotations) in ground truth data and after DeepSom classification. For the ground truth data, the Fisher's test p-value corresponds to the evidence that the proportion of somatic variants located in CGC genes differs from the corresponding proportion of non-somatic variants. For most of the datasets, DeepSom is able to reproduce a higher fraction of somatic variants in CGC genes compared to that of non-somatic ones.

dataset	GACA-CN		BLCA-US		ESAD-UK		LINC-JP		TCGA-LAML	
	SNP	INDEL	SNP	INDEL	SNP	INDEL	SNP	INDEL	SNP	INDEL
som vs germ&art	0.95	0.98	0.92	0.96	0.92	0.97	0.92	0.96	0.99	0.99
som vs germ	0.91	0.97	0.89	0.95	0.81	0.93	0.90	0.94	0.99	0.99
som vs art	0.94	0.98	0.89	0.95	0.89	0.95	0.89	0.95	0.97	0.95

Table S9. CNN output thresholds maximizing the DeepSom f1-score when separating somatic and non-somatic variants, somatic and germline variants, somatic variants and artefacts. See Section 2.6 on how the output thresholds are calculated.

dataset	DeepSom						SomVarIUS			ISOWN
	SNP			INDEL			SNP			SNP
	som vs germ &art	som vs germ	som vs art	som vs germ &art	som vs germ	som vs art	som vs germ &art	som vs germ	som vs art	som vs germ
GACA-CN	0.37	0.59	0.40	0.16	0.35	0.17	0.072	0.24	0.091	0.24
BLCA-US	0.43	0.57	0.52	0.17	0.30	0.26	0.044	0.082	0.086	0.76
ESAD-UK	0.54	0.68	0.60	0.28	0.45	0.29	0.12	0.33	0.15	0.36
LINC-JP	0.27	0.40	0.37	0.20	0.31	0.26	0.020	0.073	0.027	0.45
TCGA-LAML	0.020	0.032	0.055	0.072	0.076	0.63	5.2e-4	2.4e-3	8.6e-4	0.15

Table S10. Precision for DeepSom, SomVarIUS, and ISOWN. The table contains the mean average of precision computed separately on each sample (patient). The corresponding DeepSom CNN output threshold values can be found in Table S9.

dataset	DeepSom						SomVarIUS			ISOWN
	SNP			INDEL			SNP			SNP
	som vs germ &art	som vs germ	som vs art	som vs germ &art	som vs germ	som vs art	som vs germ &art	som vs germ	som vs art	som vs germ
GACA-CN	0.52	0.63	0.56	0.30	0.41	0.36	0.37	0.36	0.42	1.0e-3
BLCA-US	0.56	0.62	0.63	0.33	0.39	0.41	0.036	0.036	0.036	1.5e-3
ESAD-UK	0.59	0.70	0.62	0.33	0.52	0.43	0.22	0.22	0.22	9.1e-4
LINC-JP	0.43	0.51	0.54	0.29	0.41	0.34	0.047	0.047	0.047	1.2e-3
TCGA-LAML	0.15	0.15	0.34	0.13	0.13	0.42	0.035	0.019	0.037	1.8e-3

Table S11. Recall for DeepSom, SomVarIUS, and ISOWN. The table contains the mean average of recall computed separately on each sample (patient). The corresponding DeepSom CNN output threshold values can be found in Table S9.

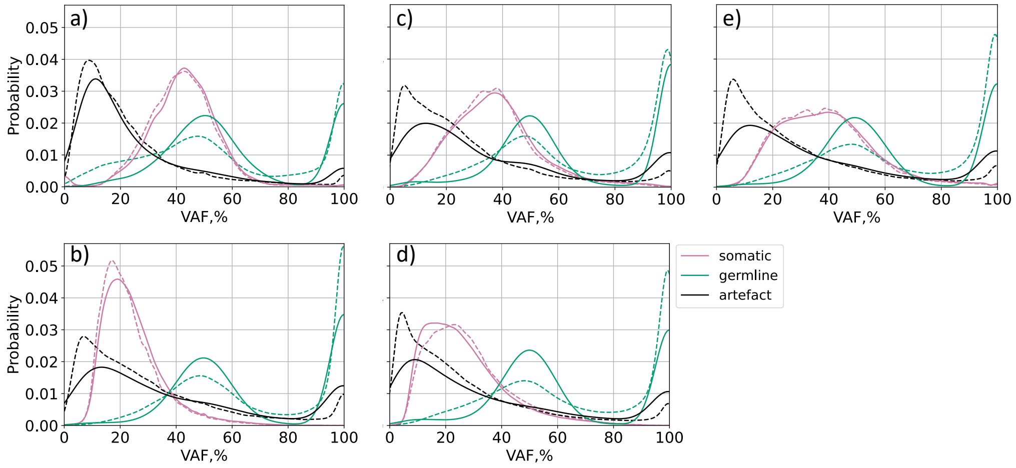

Figure S1. Variant allele fraction (VAF) distributions before (solid) and after (dashed) removing gnomAD variants for sequencing artefacts, germline variants and somatic variants in TCGA-LAML (a), GACA-CN (b), LINC-JP (c), ESAD-UK (d), and BLCA-US (e). The slight shifts of germline and artefact distributions after gnomAD filtering is probably due to false labelling of some germline variants.

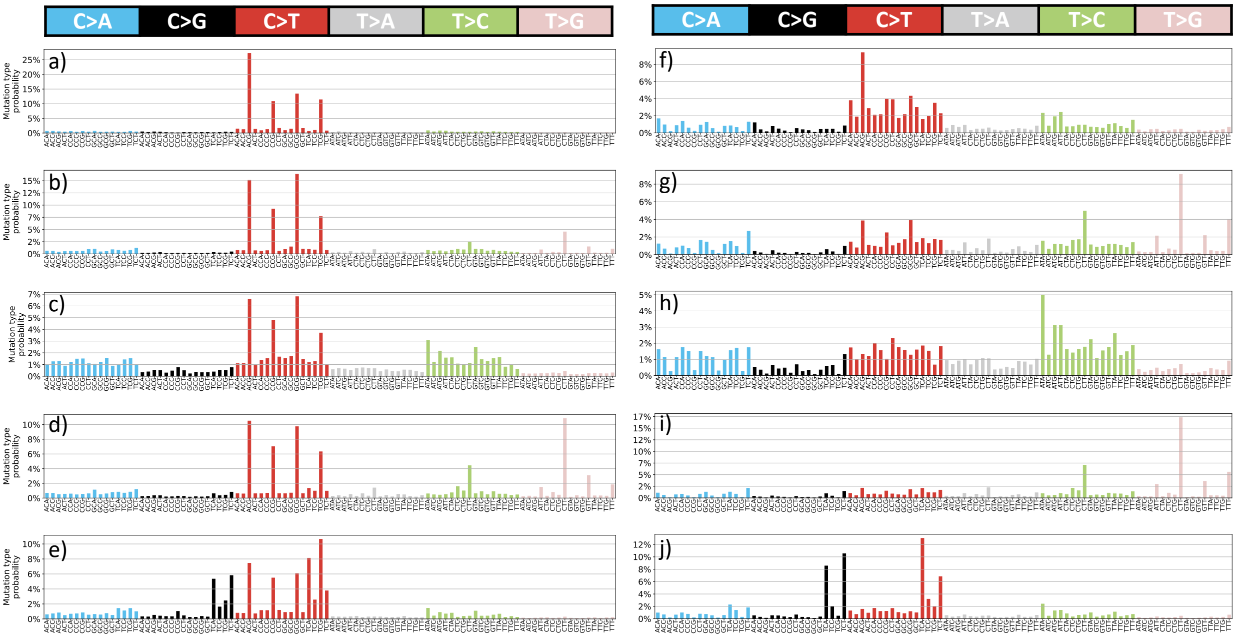

Figure S2. Normalized (a-e) and non-normalized (f-j) mutational signatures of somatic variants in TCGA-LAML (a, f), GACA-CN (b, g), LINC-JP (c, h), ESAD-UK (d, i), BLCA-US (e, j).

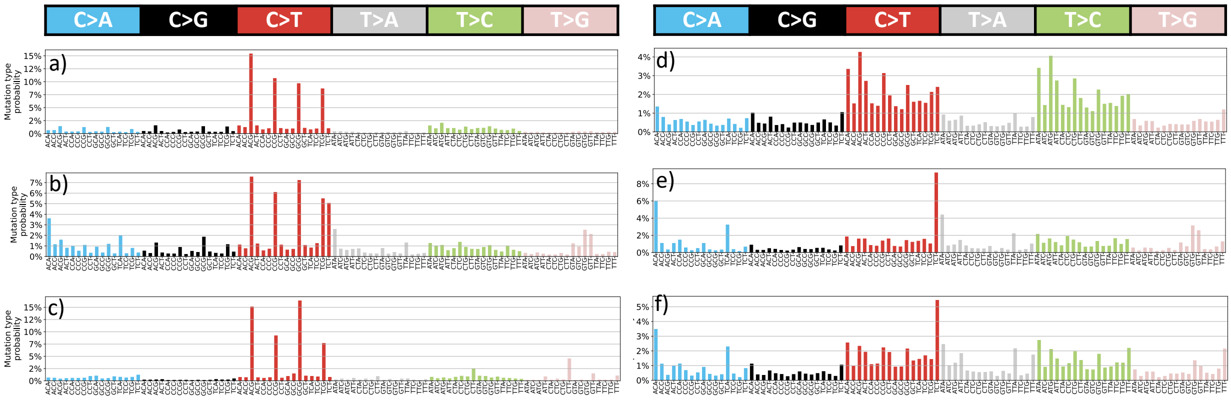

Figure S3. Normalized (a-c) and non-normalized (d-f) mutational signatures of germline variants (a, d), sequencing artefacts in the TCGA-LAML dataset (b, e), and sequencing artefacts in the ICGC datasets (c, f). Different sequencing equipment result in different artefacts signatures.

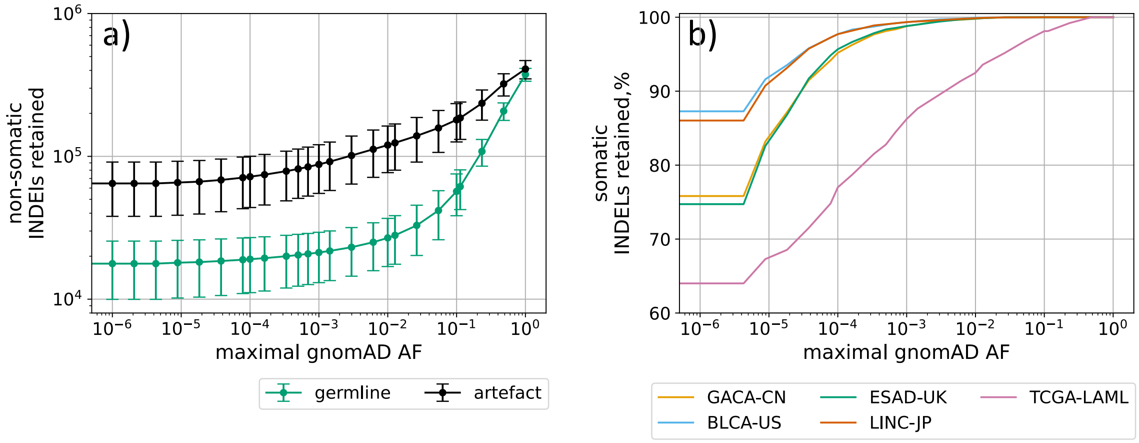

Figure S4. (a) The number of germline and artefact INDELs retained at different gnomAD allele frequency (AF) cutoffs. The errorbars show the standard deviation over all samples in all datasets. (b) The fraction of somatic INDELs retained at different gnomAD AF cutoffs in each dataset.

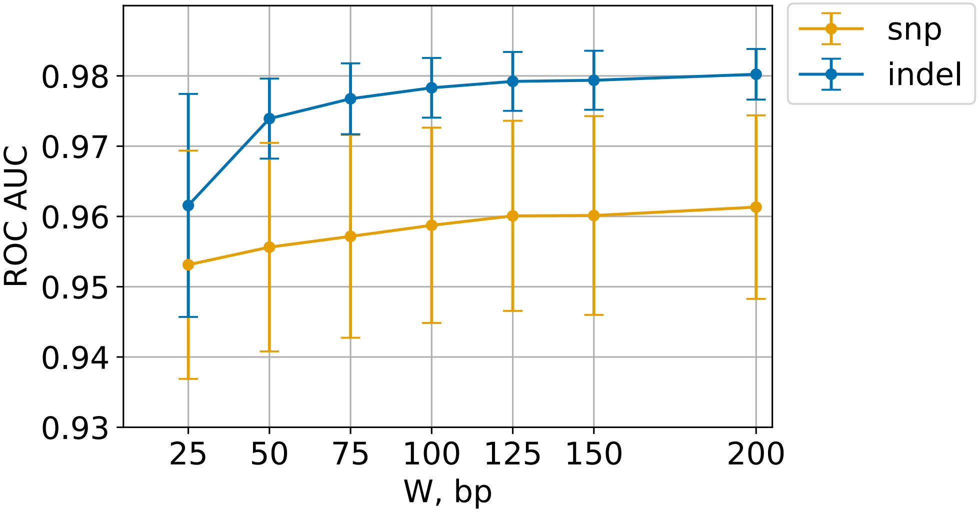

Figure S5. CNN performance on SNP and INDEL classification at different ROI length *W* for the BLCA-US dataset.

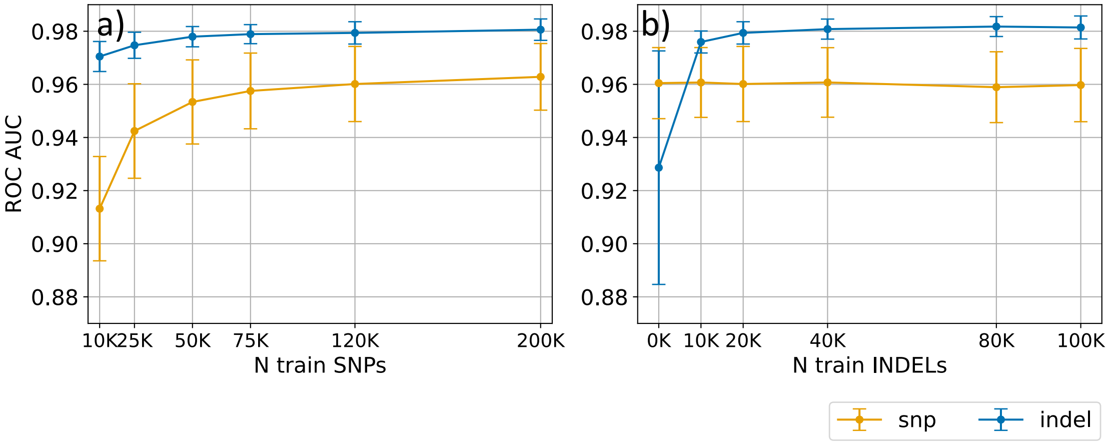

Figure S6. CNN performance on SNP and INDEL classification as a function of the number of train variants in the BLCA-US dataset. (a) varying the number of train SNPs with a fixed number of train INDELs = 20K. (b) varying the number of train INDELs with a fixed number of train SNPs = 120K.

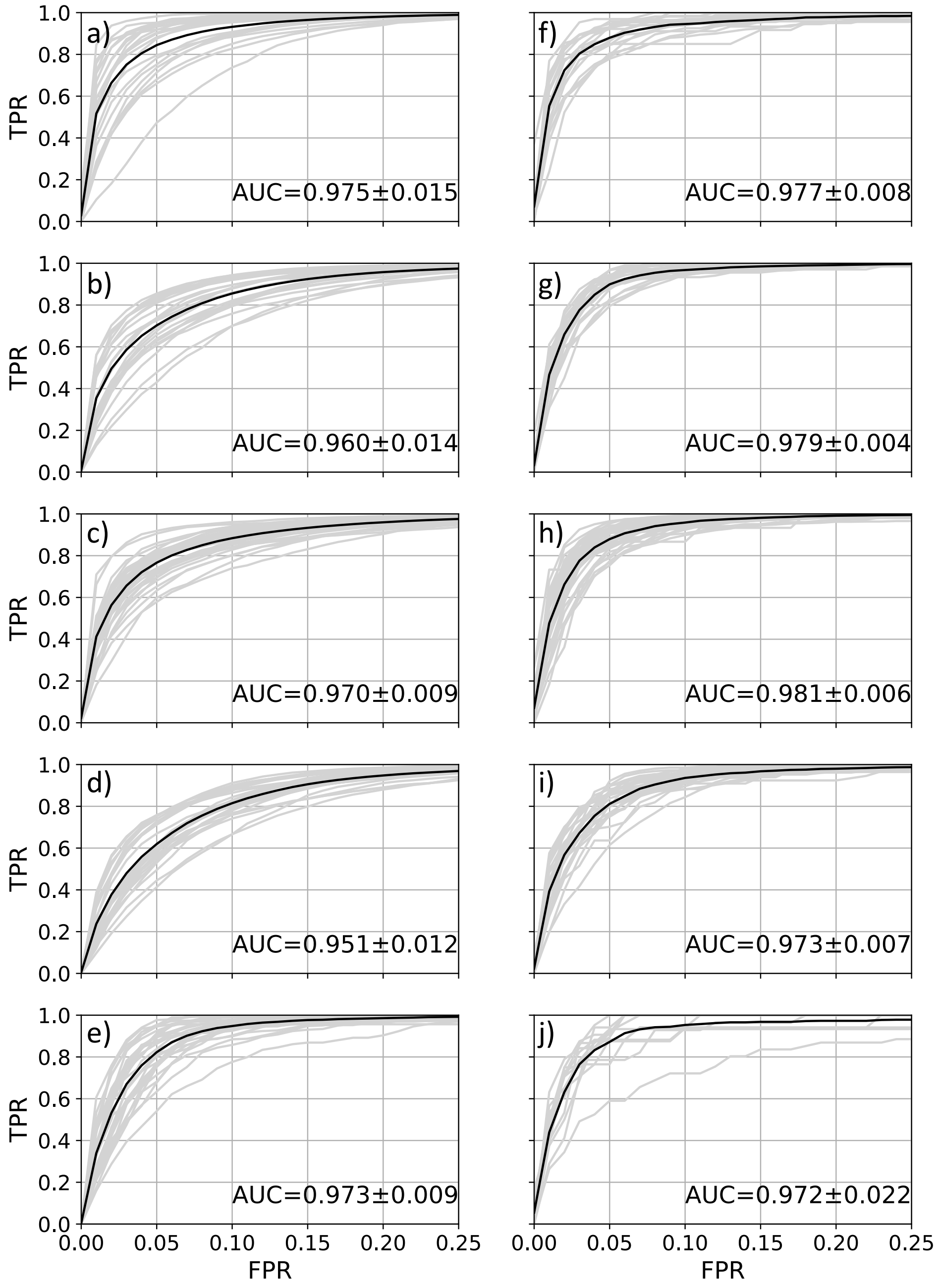

Figure S7. ROC curves for SNP (a-e) and INDEL (f-j) variants in GACA-CN (a,f), BLCA-US (b,g), ESAD-UK (c,h), LINC-JP (d,i), and TCGA-LAML (e,j). Each gray line represents a ROC curve computed on a single test sample (patient). The black line corresponds to the average ROC curve over all individual ROC curves. Samples with less than 10 ground truth somatic or less than 10 ground truth non-somatic variants are excluded. The average over all individual ROC AUC is shown in the lower right corner of each subplot.

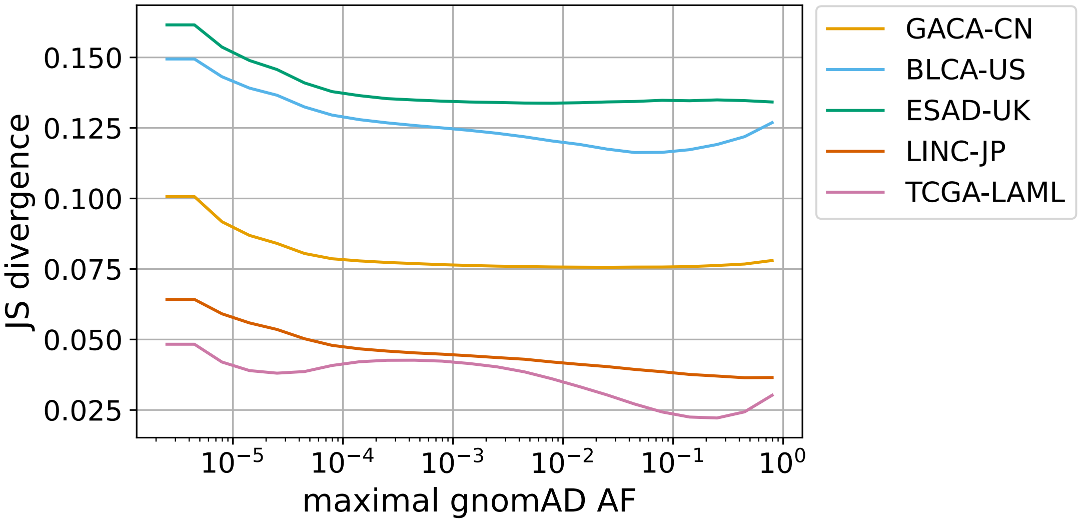

Figure S8. Jensen-Shannon (JS) divergence between somatic and germline mutational signatures as a function of the gnomAD allele frequency (AF) threshold for each dataset. The JS divergence decreases with the increase of the gnomAD threshold, indicating that germline and somatic mutational signatures become more similar.

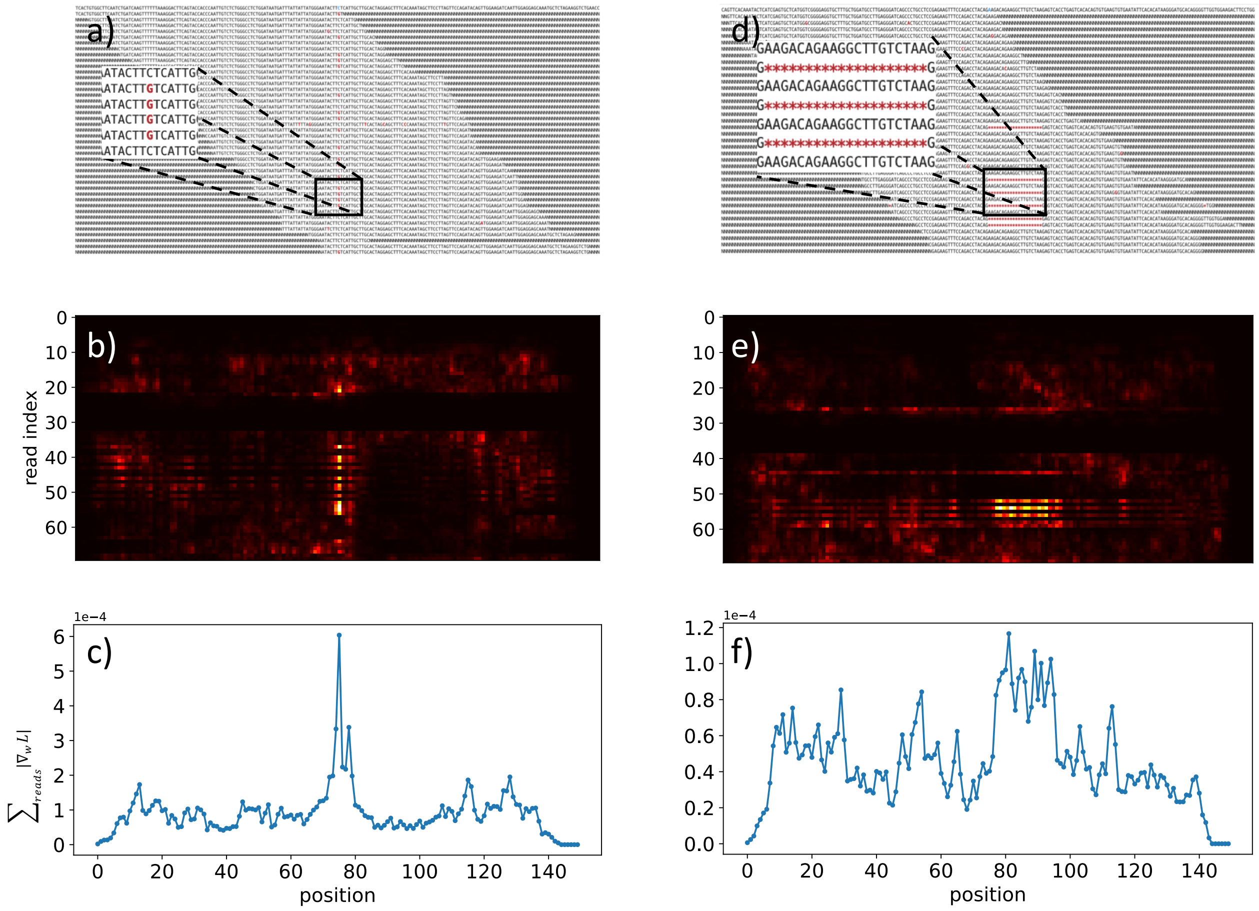

Figure S9. CNN saliency maps for a SNP (a-c) and a DEL (d-f) variants from the BLCA-US dataset. (a,d) –pileup images, (b,e) – saliency maps, brighter regions corresponding to higher gradient amplitudes, (d,f) – sum of (b,e) over the reads axis. Larger gradient amplitudes are observed for a 5-10bp active window around the SNP variant and a 19bp active window around the DEL variant.

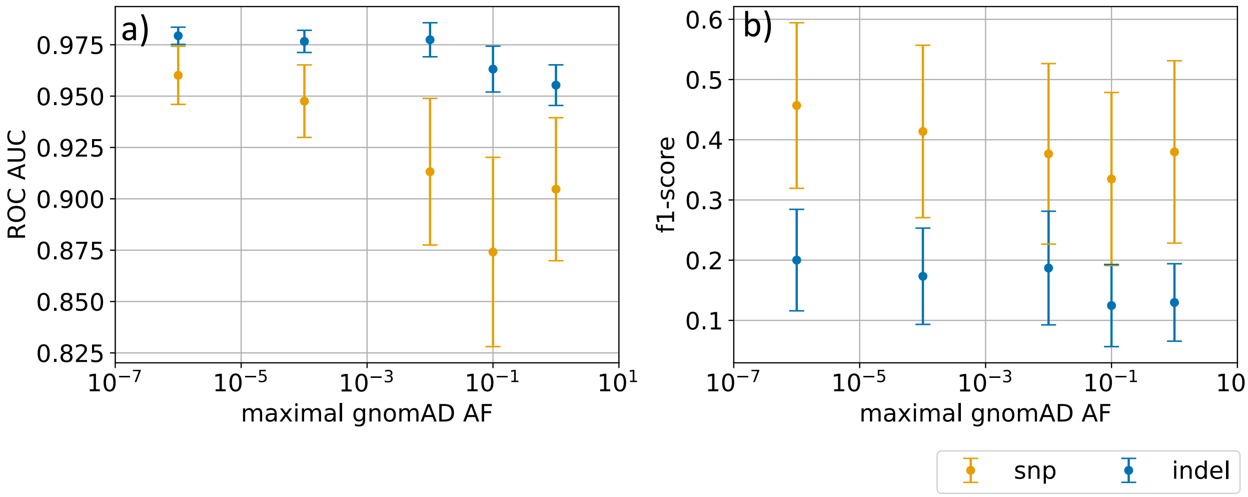

Figure S10. CNN ROC AUC score (a) and DeepSom f1-score (b) on SNP and INDEL classification at different gnomAD AF thresholds on the BLCA-US dataset. Both ROC AUC score and f1-score decrease for high gnomAD AF thresholds.

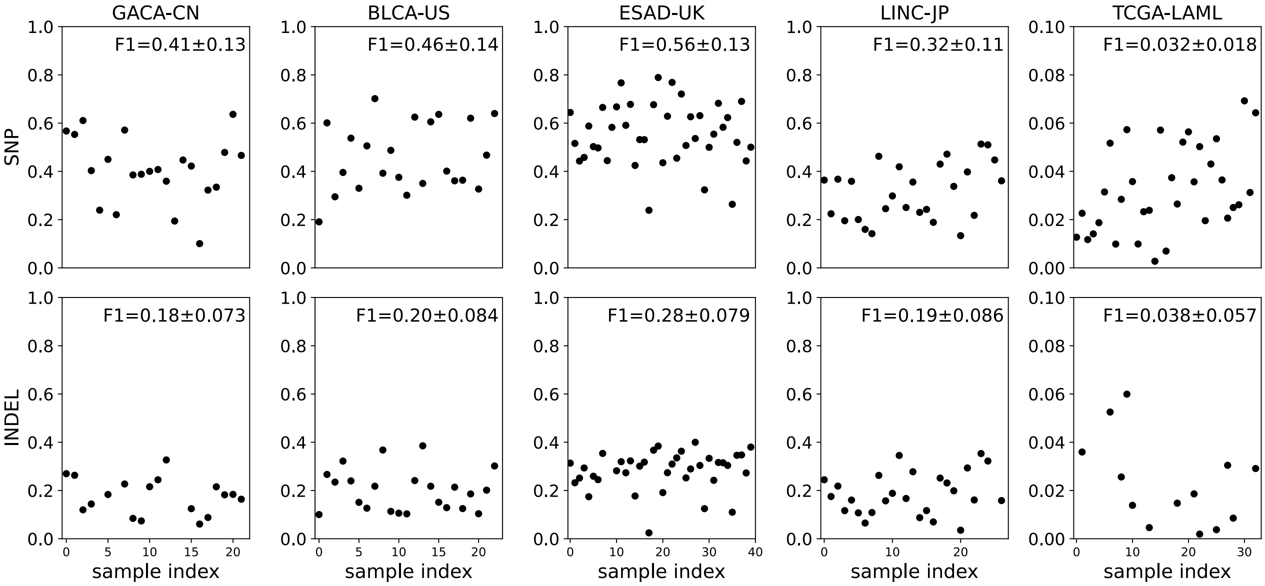

Figure S11. Per-sample f1-scores for SNP and INDEL variants in each dataset. Within each dataset, a single CNN output threshold was used to compute the f1-score for all samples (Table S9, *som vs germ&art*). Samples with less than 10 ground truth somatic or less than 10 ground truth non-somatic variants are excluded. The mean average and standard deviation over per-sample f1-scores are shown in the upper right corner of each subplot. Large per-sample f1-score fluctuations are observed due to the highly varying number of true positives.
